# From traditional to modern: easing students transition to problem-based learning

**DOI:** 10.3402/meo.v19.25438

**Published:** 2014-08-06

**Authors:** Faraz Mughal, Alice Parsloe, Rachel Stores, Jennifer Clark, Alexander Ryan, Helena Lee, Catherine Barton

**Affiliations:** 1 Public Health, Epidemiology and Biostatistics Unit, School of Health and Population Sciences, University of Birmingham, Birmingham, United Kingdom; 2 School of Health and Population Sciences, University of Birmingham, Birmingham, United Kingdom

Problem-based learning (PBL) is an innovative and contemporary method of learning that has become more prevalent within health professions education, with some medical schools in the United Kingdom (UK) now teaching a fully integrated PBL curriculum (1). This approach combines learning with the development of inter-personal skills in a small group setting (2), and evidence suggests that PBL contributes to superior student examination performance when compared to a traditional, lecture-based pedagogy (3, 4).

In 2012, we conducted a cross-sectional study of 260 first-year medical students at the University of Birmingham, where both PBL and traditional teaching methods are employed. An anonymous questionnaire was used to explore the students’ preferred teaching methodology, resources utilised, and learning behaviours.

Prior to entering medical school, virtually all (99%) students had been taught by traditional methods during UK secondary school education; of these, over half (59%) did not feel prepared for university medical education. Not surprisingly, the vast majority (97%) preferred traditional teaching over PBL—citing more structure and greater clarity. The Internet was the most common resource used in PBL study, whereas textbooks were the most used resource in traditional learning.

Despite the growing integration of PBL into medical school curricula, our first-year students strongly preferred the more traditional approach to learning with which they were familiar. While PBL provides a platform for students to develop responsibility for managing their own learning needs, a more holistic approach might be delivered via the phased integration of PBL into medical school curricula—allowing for the gradual acquisition of new learning methods. Preliminary findings suggest that implementing a combination of both PBL and lecture-based teaching can be quite well received by students (5).

Any use of PBL must include a concerted effort to establish a positive ethos with clearly stated objectives from which students can build a foundation for lifelong learning. However, recognising the positive aspects of more traditional approaches, and incorporating these within the modern PBL method, may be the optimal strategy to achieve a harmonious transition from passive to active learning.

*Faraz Mughal* Public Health, Epidemiology and Biostatistics Unit School of Health and Population Sciences University of Birmingham Birmingham, United Kingdom Email: farazm@doctors.org.uk *Alice Parsloe* School of Health and Population Sciences University of Birmingham Birmingham, United Kingdom *Rachel Stores* School of Health and Population Sciences University of Birmingham Birmingham, United Kingdom *Jennifer Clark* School of Health and Population Sciences University of Birmingham Birmingham, United Kingdom *Alexander Ryan* School of Health and Population Sciences University of Birmingham Birmingham, United Kingdom *Helena Lee* School of Health and Population Sciences University of Birmingham Birmingham, United Kingdom *Catherine Barton* School of Health and Population Sciences University of Birmingham Birmingham, United Kingdom 
